# Effects of Methyl Jasmonate on Flavonoid Accumulation and Physiological Metabolism in Finger Millet (*Eleusine coracana* L.) Sprouts

**DOI:** 10.3390/plants14142201

**Published:** 2025-07-16

**Authors:** Zhangqin Ye, Jing Zhang, Xin Tian, Zhengfei Yang, Jiangyu Zhu, Yongqi Yin

**Affiliations:** College of Food Science and Engineering, Yangzhou University, Yangzhou 225009, China; mz120242240@stu.yzu.edu.cn (Z.Y.); mz120222090@stu.yzu.edu.cn (J.Z.); dx120230241@stu.yzu.edu.cn (X.T.); yzf@yzu.edu.cn (Z.Y.); 008051@yzu.edu.cn (J.Z.)

**Keywords:** flavonoid, finger millet sprouts, methyl jasmonate

## Abstract

Finger millet (*Eleusine coracana* L.) is a nutrient-dense cereal with high flavonoid content, yet the mechanisms regulating its secondary metabolite biosynthesis remain underexplored. Various exogenous stimuli can readily activate the enzymatic pathways and gene expression associated with flavonoid biosynthesis in plants, which are regulated by developmental cues. Research has established that methyl jasmonate (MeJA) application enhances secondary metabolite production in plant systems. This investigation examined MeJA’s influence on flavonoid accumulation and physiological responses in finger millet sprouts to elucidate the molecular mechanisms underlying MeJA-mediated flavonoid accumulation. The findings revealed that MeJA treatment significantly suppressed sprout elongation while enhancing the biosynthesis of total flavonoids and phenolic compounds. MeJA treatment triggered oxidative stress responses, with hydrogen peroxide and superoxide anion concentrations increasing 1.84-fold and 1.70-fold compared to control levels at 4 days post-germination. Furthermore, the antioxidant defense mechanisms in finger millet were upregulated following treatment, resulting in significant enhancement of catalase and peroxidase enzymatic activities and corresponding transcript abundance. MeJA application augmented the activities of key phenylpropanoid pathway enzymes—phenylalanine ammonia-lyase (PAL) and cinnamate 4-hydroxylase (C4H)—and upregulated their respective gene expression. At 4 days post-germination, *EcPAL* and *EcC4H* transcript levels were elevated 3.67-fold and 2.61-fold, respectively, compared to untreated controls. MeJA treatment significantly induced the expression of downstream structural genes and transcriptional regulators. This study provides a deeper understanding of the mechanism of flavonoid accumulation in foxtail millet induced by MeJA, and lays a foundation for exogenous conditions to promote flavonoid biosynthesis in plants.

## 1. Introduction

Flavonoids, which are secondary metabolites synthesized through the phenylpropanoid pathway in plants, are characterized by hydroxyl groups and conjugated double-bond systems in their molecular structures [1]. These structural features are known to confer exceptional free radical scavenging capacity, effectively mitigating cellular oxidative damage induced by reactive oxygen species (ROS) [2,3]. Substantial clinical and epidemiological evidence shows that regularly consuming adequate amounts of flavonoids can significantly improve human health through various protective mechanisms. Research indicates that moderate flavonoid intake protects cardiovascular health by reducing risks of vascular diseases, including atherosclerosis and hypertension, mainly by improving endothelial function and optimizing lipid balance [4]. These compounds also promote metabolic health by lowering the incidence of type 2 diabetes, an effect attributed to their ability to enhance insulin sensitivity and maintain glucose homeostasis [5]. Furthermore, flavonoids exhibit notable anticancer potential, particularly against hormone-related malignancies, including breast and prostate cancers, as they can modulate estrogen receptor activity and inhibit tumor cell proliferation [6,7]. These cumulative findings underscore the importance of incorporating dietary flavonoids as part of a balanced nutritional strategy for chronic disease prevention [8]. However, flavonoids cannot be endogenously synthesized by humans and must be acquired through dietary sources. Consequently, the development of flavonoid-enriched foods has gained increasing global attention.

Finger millet (*Eleusine coracana* L.), an annual cereal crop belonging to the *Poaceae* family, is native to Ethiopia in Africa and has been introduced to various regions in Asia [9]. In China, it is primarily cultivated in the southwestern regions [10]. Beyond its nutritional value as a staple crop, finger millet has gained increasing attention for its rich flavonoid content and associated health benefits. Epidemiological and clinical studies have documented its therapeutic potential, including anti-atherosclerotic [11], anti-diabetic [12], and anti-tumor activities [13]. Long-term consumption of finger millet has been reported to inhibit low-density lipoprotein oxidation, alleviate hypertension, hypercholesterolemia, and diabetes, and improve gastrointestinal function and vascular integrity [14,15]. To investigate the mechanisms underlying flavonoid biosynthesis, this study employed methyl jasmonate (MeJA) as an elicitor in finger millet sprouts. During plant growth stages, the application of phytohormones has been proven to effectively promote the accumulation of secondary metabolites such as flavonoids in sprouts [16].

Research indicates that methyl jasmonate (MeJA), as a plant hormone, relies on the jasmonic acid (JA) metabolic pathway within plants for its endogenous synthesis [17]. Endogenous MeJA levels are regulated by multiple factors, including the expression of synthesis-related genes, induction by external signals, and regulation through epigenetic modifications [18]. MeJA performs various functions in plant physiological processes, particularly in stress resistance and defense metabolism regulation, where it can activate key enzyme genes in secondary metabolic pathways to promote the biosynthesis of defensive secondary metabolites in plants [19,20]. Recent studies have demonstrated that preharvest application of MeJA significantly influences secondary metabolite production in various berry crops. In raspberry plants, foliar MeJA treatment has been shown to induce a marked increase in flavonoid content, particularly quercetin and kaempferol derivatives, as reported by [21]. This phytohormonal elicitation effect extends to other *Rubus* species [22], with experimental evidence indicating that MeJA treatment not only elevates flavonoid concentrations but also substantially enhances the antioxidant capacity in blackberry fruit [23]. The regulatory role of MeJA in phenylpropanoid pathway activation appears particularly pronounced in *Vitis* species. Research findings reveal that exogenous MeJA application stimulates anthocyanin biosynthesis in grape berries, resulting in significantly higher pigment accumulation in both the skin and flesh tissues [24]. Similarly, MeJA treatment has been observed to upregulate key anthocyanin synthesis genes, leading to enhanced coloration and improved phytochemical profiles in strawberry fruits [25]. Germination experiments in maize further confirmed that treatment with 0.15 μmol/L MeJA significantly elevated carotenoid biosynthesis gene expression, resulting in a 2.10-fold increase in free radical scavenging activity [26].

This study examined how MeJA treatment affected the growth metabolism and flavonoid accumulation in finger millet sprouts. Furthermore, by examining the expression of genes encoding antioxidant and flavonoid biosynthesis pathway enzymes, the regulatory mechanisms behind finger millet’s reaction to MeJA treatment were clarified. For future studies on MeJA-mediated flavonoid production in finger millet sprouts, our results offer a theoretical basis.

## 2. Results

### 2.1. Effects of MeJA Treatment on the Total Phenolics and the Total Flavonoid Content of Finger Millet Sprouts

**Figure 1:**
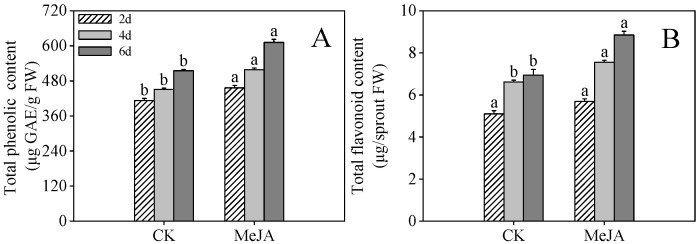
Effects of MeJA treatment on total phenolic content (**A**) and total flavonoid content (**B**) in finger millet sprouts. Lowercase letters mark significant differences between treatments within the same germination period (one-way ANOVA with Tukey’s test, *p* < 0.05) 2d: two days after germination; 4d: four days after germination; 6d: six days after germination.

As shown in Figure 1A, MeJA treatment significantly increased phenolic compound production in finger millet sprouts (*p* < 0.05). MeJA-treated samples exhibited 1.11-, 1.15-, and 1.19-fold higher total phenolic concentrations compared to control samples at 2, 4, and 6 days after imbibition, respectively (Figure 1A). Total flavonoid levels increased steadily throughout germination, with MeJA-treated samples at 4 and 6 days after imbibition containing 1.14- and 1.28-fold higher concentrations than untreated controls (Figure 1B).

### 2.2. Effects of MeJA Treatment on the Growth and Development of Finger Millet Sprouts

**Figure 2:**
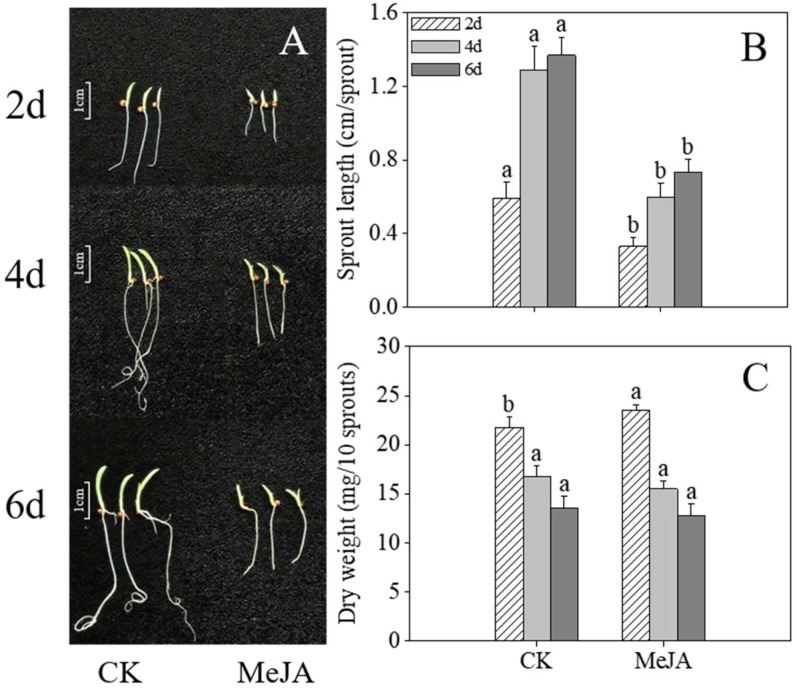
Effects of MeJA treatment on morphology (**A**), sprout length (**B**), and dry weight (**C**) in finger millet sprouts. Lowercase letters mark significant differences between treatments within the same germination period (one-way ANOVA with Tukey’s test, *p* < 0.05). 2d: two days after germination; 4d: four days after germination; 6d: six days after germination.

Compared to the control, MeJA-treated finger millet sprouts exhibited retarded development at 4 and 6 days after germination (Figure 1A), although no significant difference in dry weight was observed (Figure 2C, *p* > 0.05). MeJA treatment caused severe growth inhibition, with sprout length significantly shorter than in the control group (Figure 2B, *p* < 0.05). While root development appeared sturdier in treated sprouts, shoot development was markedly delayed, particularly in sprouts germinated for over 2 days.

### 2.3. Effects of MeJA Treatment on the Antioxidant Capacity of Finger Millet Sprouts

**Figure 3:**
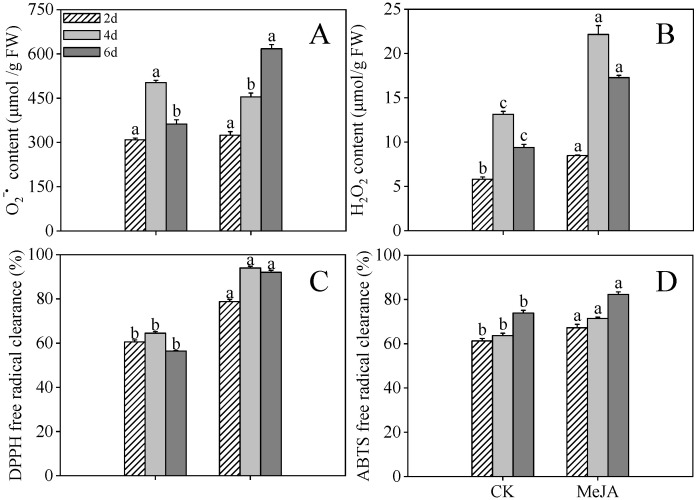
Effects of MeJA treatment on O_2_^−•^ (**A**), H_2_O_2_ (**B**), DPPH radical scavenging capacity (**C**), and ABTS free radical scavenging capacity (**D**) of finger millet sprouts. Lowercase letters mark significant differences between treatments within the same germination period (one-way ANOVA with Tukey’s test, *p* < 0.05). 2d: two days after germination; 4d: four days after germination; 6d: six days after germination.

As depicted in Figure 3A, the O_2_^−•^ concentrations in MeJA-treated finger millet sprouts increased progressively throughout the germination phase (*p* < 0.05, one-way ANOVA), attaining peak values 1.70-fold greater than control cohorts at 6 days post-imbibition. MeJA-treated specimens maintained significantly elevated H_2_O_2_ levels compared to controls at 2 and 4 days post-germination (1.69-fold and 1.84-fold, respectively, Figure 3B, *p* < 0.05). The observed accumulation of O_2_^−•^ and H_2_O_2_ indicates ROS overproduction and consequent oxidative cellular impairment under MeJA treatment conditions.

Additionally, DPPH scavenging activity reached maximum efficacy in 4-day post-imbibition specimens (Figure 3C), exhibiting 1.46-fold enhancement compared to controls, while ABTS scavenging potential peaked at 6 days post-imbibition (11.37% elevation relative to controls, Figure 3D). These findings substantiate that MeJA administration substantially augmented the antioxidant defense mechanisms in finger millet sprouts.

### 2.4. Effects of MeJA Treatment on Activity and Gene Expression Level of Antioxidant Enzyme in Finger Millet Sprouts

**Figure 4:**
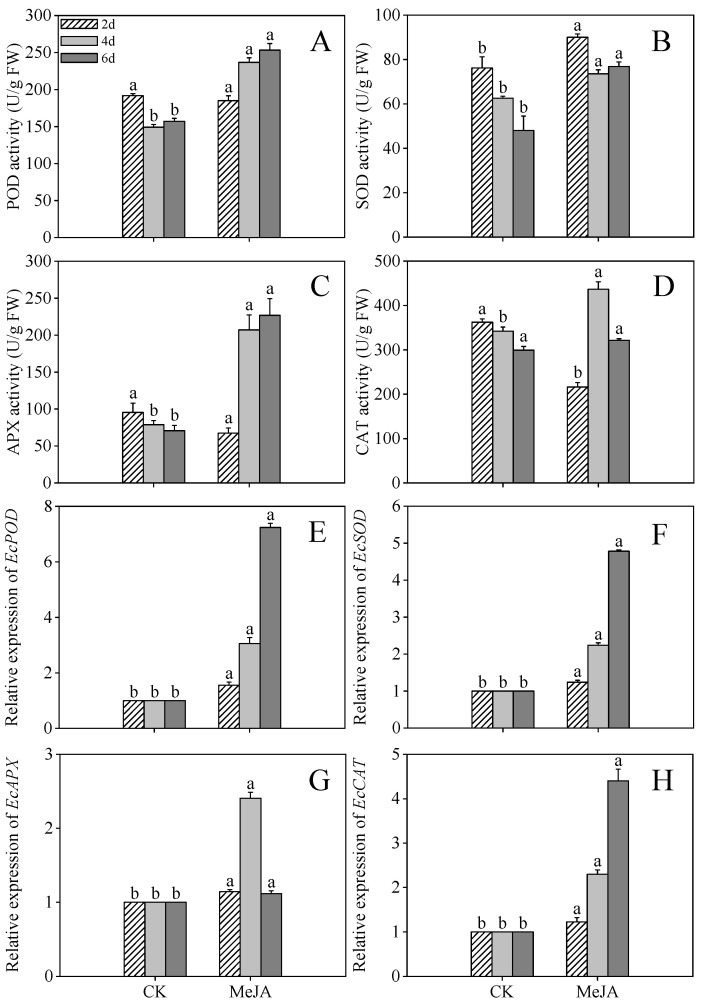
Effects of MeJA treatment on peroxidase (POD) activity (**A**), superoxide dismutase (SOD) activity (**B**), ascorbate peroxidase (APX) activity (**C**), catalase (CAT) activity (**D**), and the gene expression levels of *EcPOD* (**E**), *EcSOD* (**F**), *EcAPX* (**G**), and *EcCAT* (**H**) of finger millet sprouts. Lowercase letters mark significant differences between treatments within the same germination period (one-way ANOVA with Tukey’s test, *p* < 0.05). 2d: two days after germination; 4d: four days after germination; 6d: six days after germination.

Figure 4A,C illustrate that the highest POD (253.38 U/g) and APX (226.87 U/g) activities were observed in 6-day post-germination sprouts, representing 1.61- and 3.20-fold increases over the control. CAT activity peaked at 436.70 U/g (Figure 4D, 1.28-fold of the control) in 4-day post-germination sprouts, while SOD activity reached 90.03 U/g (Figure 4B, 1.18-fold of the control) at 2 days post-germination. These findings indicate that finger millet sprouts alleviate stress damage by modulating POD, SOD, APX, and CAT activities.

The relative expression levels of antioxidant enzyme genes (*EcCAT*, *EcPOD*, *EcSOD*, and *EcAPX)* under MeJA treatment are shown in Figure 4E–H. MeJA treatment significantly increased the expression of *EcCAT*, *EcPOD*, and *EcSOD* in 4- and 6-day post-germination sprouts compared to the control, while *EcAPX* expression in 4-day post-germination sprouts was elevated 2.25-fold compared to the control group (*p* < 0.05). These results suggest that oxidative stress induced by growth constraints triggers enhanced antioxidant enzyme activity as a compensatory mechanism.

### 2.5. Effects of MeJA Treatment on Activity and Gene Expression Level of Key Enzymes Associated with Flavonoid Biosynthesis in Finger Millet Sprouts

**Figure 5:**
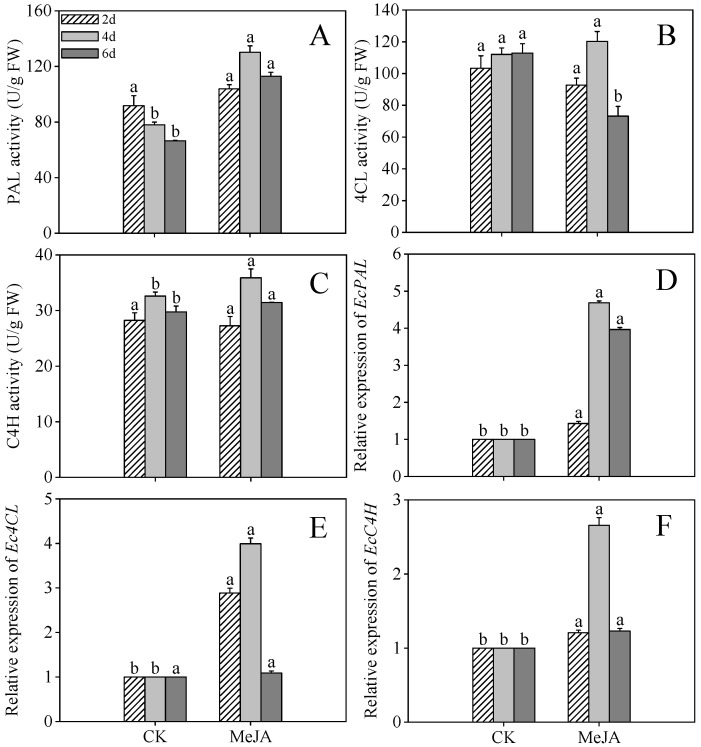
Effects of MeJA treatment on phenylalanine ammonia-lyase (PAL) activity (**A**), 4-coumarate-CoA ligase (4CL) activity (**B**), cinnamate 4-hydroxylase (C4H) activity (**C**), and the gene expression levels of *EcPAL* (**D**), *Ec4CL* (**E**), and *EcC4H* (**F**) of finger millet sprouts. Lowercase letters mark significant differences between treatments within the same germination period (one-way ANOVA with Tukey’s test, *p* < 0.05). 2d: two days after germination; 4d: four days after germination; 6d: six days after germination.

As illustrated in Figure 5A,C, by day 4 post-germination, PAL and C4H activities in MeJA-elicited sprouts reached maximal values of 130.17 U/g and 35.91 U/g, constituting 1.67-fold and 1.10-fold enhancements compared to untreated controls, respectively. However, 4CL activity exhibited no statistically significant variation between MeJA-treated and control sprouts at both 2 and 4 days following germination. 4CL activity in MeJA-treated sprouts decreased markedly by 54.19% compared to the control at 6 days post-germination. Data analysis revealed that the heightened flavonoid levels detected in MeJA-treated sprouts directly result from increased activity of essential biosynthetic enzymes within the phenolic pathway.

MeJA treatment significantly upregulated the relative expression of *EcPAL*, *Ec4CL*, and *EcC4H* in 4-day post-germination sprouts (Figure 5D–F, *p* < 0.05), increasing 3.67-, 3.98-, and 2.61-fold compared to the control, respectively. The concordance between enzyme activity and gene expression highlights the critical role of transcriptional regulation in phenolic compound accumulation.

### 2.6. Effects of MeJA Treatment on Gene Expression in the Middle and Downstream Pathways of Flavonoid Metabolism in Finger Millet Sprouts

**Figure 6:**
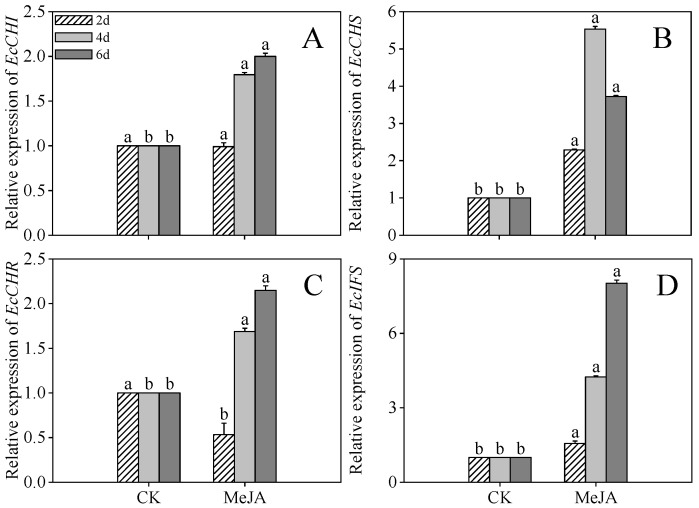
The effects of MeJA treatment on *EcCHI* (**A**), *EcCHS* (**B**), *EcCHR* (**C**), and *EcIFS* (**D**) relative expression of finger millet sprouts. Lowercase letters mark significant differences between treatments within the same germination period. CHI: chalcone isomerase; CHS: chalcone synthase; CHR: chalcone reductase; IFS: isoflavone synthase (one-way ANOVA with Tukey’s test, *p* < 0.05). 2d: two days after germination; 4d: four days after germination; 6d: six days after germination.

Compared to the control, MeJA-treated 4-day post-germination sprouts exhibited 0.79-, 4.43-, 0.68-, and 3.22-fold higher expression of *EcCHI*, *EcCHS*, *EcCHR*, and *EcIFS*, respectively. Elevated expression of these genes persisted in 6-day post-germination sprouts under MeJA treatment. In contrast, *EcCHS* expression initially increased but subsequently declined (Figure 6B).

### 2.7. Effects of MeJA Treatment on Stress-Related Gene Expression in Finger Millet Sprouts

**Figure 7:**
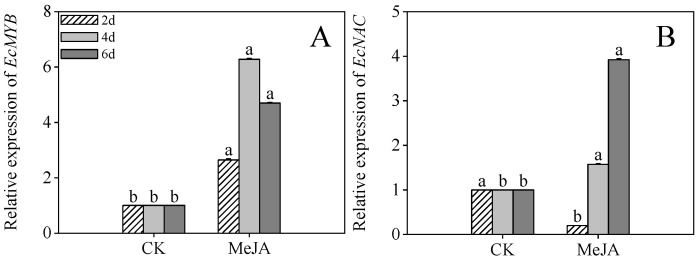
The effects of MeJA treatment on *EcMYB* (**A**) and *EcNAC* (**B**) relative expression of finger millet sprouts. Lowercase letters mark significant differences between treatments within the same germination period (one-way ANOVA with Tukey’s test, *p* < 0.05). 2d: two days after germination; 4d: four days after germination; 6d: six days after germination.

Figure 7A revealed that MeJA treatment significantly enhanced *EcMYB* expression in 4- and 6-day post-germination sprouts (6.27- and 4.69-fold compared to the control, respectively, *p* < 0.05). Similarly, *EcNAC* expression in 6-day post-germination sprouts was 3.91-fold higher than the control (Figure 7B, *p* < 0.05). These findings suggest that the transcription factors *EcMYB* and *EcNAC* play pivotal roles in mitigating stress during the mid-to-late germination stages.

## 3. Discussion

During germination, environmental stress induces ROS generation, potentially reducing the nutritional value of food. Plants counter this stress through antioxidant defense systems, including phenolic and flavonoid compounds synthesized via the phenylpropanoid metabolic cascade in non-enzymatic pathways. Plant hormones such as MeJA effectively activate these biosynthetic pathways. This research demonstrates that MeJA treatment significantly increased H_2_O_2_ (1.84-fold) and O_2_^−•^ (1.70-fold) concentrations on the fourth day post-germination in finger millet sprouts, while simultaneously promoting flavonoid/phenolic compound accumulation. Notably, H_2_O_2_ peaked 48 h post-treatment, preceding the maximum transcription levels of phenylpropanoid metabolism-related genes (such as *EcPAL*, upregulated 3.67-fold at 72 h), suggesting ROS may function as signaling molecules for flavonoid biosynthesis. Additionally, MeJA upregulated the activity and gene expression of POD and SOD during days 4–6 of finger millet germination, as well as CAT and APX on day 4, exhibiting a coordinated antioxidant enzyme system activation pattern. This integrated response confirms MeJA’s central role in regulating plant oxidative stress defense networks, consistent with findings from germinating peanut studies [27,28].

Morphologically, MeJA treatment significantly reduced sprout elongation at 4 and 6 days post-germination, likely attributable to its function as an endogenous signaling molecule that activates plant defense mechanisms, including enhanced stress tolerance, regulated growth, secondary metabolite synthesis via signal transduction, and improved self-repair capacity [28]. Analogous to our observations, previous research demonstrated that MeJA treatment inhibited peanut sprout growth and induced resveratrol accumulation [27]. Under abiotic stress conditions, plants frequently accumulate soluble sugars for osmotic adjustment, thereby adapting to environmental perturbations [29].

The flavonoid biosynthetic pathway initiates via the phenylpropanoid pathway, with PAL, C4H, and 4CL functioning as key enzymatic regulators. To elucidate the mechanism underlying flavonoid enrichment in finger millet sprouts, we assessed these enzyme activities. MeJA treatment significantly enhanced PAL activity at 4 and 6 days post-germination and C4H activity at 4 days post-germination compared to controls, indicating that MeJA augments flavonoid synthesis by enhancing key enzymatic activities. Similar strategies for enhancing secondary metabolism have been documented, such as abscisic acid-induced PAL activation promoting phenolic acid accumulation in germinating wheat [30].

To further investigate the regulatory mechanisms, we analyzed gene expression profiles in the flavonoid biosynthetic pathway. The expression patterns of *EcPAL* and *EcC4H* in the phenylpropanoid pathway correlated with their corresponding enzyme activities under MeJA treatment, confirming that MeJA promotes flavonoid accumulation through transcriptional upregulation. Similar molecular mechanisms were observed in peanuts, where [31] MeJA treatment elevated PAL, C4H, and 4CL activities and gene expression in cotyledonous and non-cotyledonous tissues to enhance resveratrol biosynthesis [32].

In our study, chalcone isomerase (CHI), chalcone synthase (CHS), and chalcone reductase (CHR)—key enzymes in downstream flavonoid branch pathways—facilitated flavonoid conversion [33]. Isoflavone synthase (IFS), which specifically determines flavonoid production, was also investigated [34]. MeJA treatment significantly upregulated the relative expression of *EcCHI*, *EcCHS*, *EcCHR*, and *EcIFS* compared to controls. While acid-treated germinating soybeans exhibited similar upregulation of *EcCHI*, *EcCHS*, and *EcIFS*, discrepancies in *EcCHR* expression may result from treatment-specific or species-dependent variations.

MYB and NAC transcription factors are key regulators of stress adaptation and secondary metabolism [35,36]. Our data reveal that MeJA significantly upregulates *EcMYB* and *EcNAC* expression in finger millet sprouts, aligning with the reported roles of MYB factors in jasmonate-mediated flavonoid biosynthesis and NAC induction by MeJA in other species [37,38]. Future work will define the specific functions of *EcMYB/EcNAC* in stress mitigation and metabolic regulation during elicitor treatments.

MeJA-induced flavonoid enrichment demonstrates significant application value. From a nutritional perspective, millet sprouts with high flavonoid content can serve as preventive dietary supplements for cardiovascular diseases, holding particular significance in regions with limited food diversity [39,40]. From a commercial standpoint, millet sprouts show tremendous potential as high value-added functional food ingredients: market research indicates that grain products rich in bioactive compounds can command a premium price [41], while their germination-active characteristics simplify large-scale production processes. Compared to transgenic technology, this biofortification strategy circumvents regulatory barriers associated with genetically modified organisms, accelerating the product commercialization process.

## 4. Materials and Methods

### 4.1. Plant Materials and Treatments

Homogeneous, fully developed finger millet sprouts were selected and subjected to rigorous washing using distilled water for complete removal of surface particulates. Subsequent sterilization involved immersion in 1% (*v*/*v*) sodium hypochlorite solution for 10 min, with thorough rinsing in deionized water until pH neutrality was attained. Post-sterilization, samples underwent imbibition at 31 °C for 6 h before transfer to growth chambers. Germination proceeded under dark conditions at 31 °C for 6 days. The experimental design comprised two treatments: control (CK) receiving aqueous sprays and MeJA-treated samples administered 150 μM methyl jasmonate foliar applications. This concentration was optimized through preliminary trials. Nutrient solutions were replenished at 12 h intervals throughout cultivation. Germinated specimens were collected at 2-, 4-, and 6-day intervals, rapidly frozen in liquid nitrogen, and preserved at −80 °C for subsequent biochemical assays.

### 4.2. Instrumentation

The main instruments used in this study are listed in Table 1.

### 4.3. Determination of Sprout Length and Dry Weight

For each experimental condition, a randomized subset of thirty sprouts was isolated. Morphometric assessment of sprout elongation and gravimetric analysis of desiccated biomass were conducted in accordance with the methodological framework established in Ma et al.’s research [42].

### 4.4. Determination of Total Flavonoid and Total Phenolic Content

Total flavonoid extraction employed freshly harvested sprouts using 80% (*v*/*v*) ethanolic solution as the extraction solvent. This homogenate underwent ultrasonic processing (25 °C, 25 min) prior to high-speed centrifugation (8000× *g*, 10 min). The resulting supernatant was diluted 25-fold before spectrophotometric quantification at 265 nm, with genistein-based calibration enabling flavonoid quantification [43]. For phenolic analysis, sprout homogenates prepared in 50% methanol were centrifuged to obtain soluble fractions. These extracts were reacted with Folin–Ciocalteu reagent and sodium carbonate solution, incubated under light-protected conditions (25 °C, 2 h), and quantified spectrophotometrically at 765 nm against a gallic acid standard curve [44].

### 4.5. Determination of H_2_O_2_ and O_2_^−•^ Content, DPPH, and ABTS

H_2_O_2_ concentration was determined using the xylenol orange oxidation assay according to the protocol described by Zhao et al. [45]. Sprout specimens were homogenized in acidic extraction buffer (2.5 M H_2_SO_4_) containing 25 mM FeSO_4_ and 25 mM (NH_4_)_2_SO_4_. The homogenate was supplemented with 125 μM xylenol orange and 100 mM sorbitol. Following centrifugation (9000× *g*, 5 min), 100 μL of the supernatant was combined with 1 mL xylenol orange reagent. After 30 min incubation, the absorbance of the Fe^3+^ complex was measured spectrophotometrically at 560 nm.

O_2_^−•^ levels were assessed by monitoring hydroxylamine-mediated nitrite formation. Fresh sprout tissues were homogenized in 65 mM phosphate buffer (pH 7.8) and centrifuged at 8000× *g* for 10 min. A 1 mL aliquot of the supernatant was mixed with 0.9 mL phosphate buffer and 0.1 mL hydroxylammonium chloride (10 mM). Following a 20 min incubation at 25 °C, the mixture was treated with sulfanilic acid (17 mM) and α-naphthylamine (7 mM) solutions. Spectrophotometric analysis at 530 nm enabled quantification of O_2_^−•^ generation rates via calibration against a sodium nitrite standard curve.

DPPH radical scavenging activity was evaluated according to Rumpf et al. [46] by monitoring absorbance reduction at 515 nm with ascorbic acid serving as the positive control. ABTS radical scavenging capacity was determined by spectrophotometrically measuring absorbance at 734 nm.

### 4.6. Determination of Flavonoid Biosynthetic Enzyme Activities

A 0.1 M Tris-HCl buffer (pH 8.9) was utilized as the homogenization medium for sprout tissue samples. After mechanical disruption, samples underwent centrifugation at 12,000× *g* for 25 min at 4 °C to separate the pellet. The enzyme-rich supernatant was promptly collected to preserve enzymatic integrity for subsequent activity assays. PAL, C4H, and 4CL activities were quantified according to the protocol described by Ma et al. [47], with one unit of enzyme activity defined as an absorbance increase of 0.01 per min at OD_290 nm_ for PAL, OD_340 nm_ for C4H, and OD_333 nm_ for 4CL.

### 4.7. Determination of Antioxidant Enzyme Activities

The sprout samples were homogenized in an ice-cold 50 mM sodium phosphate buffer (pH 7.0) containing 1% polyvinylpyrrolidone (*v*/*v*). Following homogenization, the suspension was centrifuged at 12,000× *g* for 15 min at 4 °C, and the supernatant was subsequently collected for enzymatic assays. SOD activity was measured in units (U), with one unit defined as the amount of enzyme required to inhibit the rate of epinephrine autoxidation by 50% under specified assay conditions [48]. For APX, activity was defined as one unit per min for every 0.01 absorbance shift at 290 nm. POD and CAT activities were measured in units per gram of protein (U/g), with a single unit corresponding to a 0.01 absorbance change per min at OD_470 nm_ (POD) or OD_240 nm_ (CAT) [49].

### 4.8. RNA Extraction and Quantitative Real-Time PCR Analysis

Total RNA was extracted from cotyledon and non-cotyledon tissues utilizing a Plant RNA Isolation Kit (R6827-01, OMEGA, Norcross, GA, USA). Complementary DNA synthesis was conducted with PrimeScript^TM^ RT Master Mix (RR036A, Takara Bio, Kusatsu, Japan). Quantitative real-time polymerase chain reaction (qRT-PCR) analysis was performed using SYBR^®^ Premix Ex Taq^TM^ (RR420A, Takara, Japan) on an ABI 7500 system (Applied Biosystems, Foster City, CA, USA), with triplicate biological samples. Primer sequences are listed in Table 2. Gene expression was quantified using the comparative Ct (2^−ΔΔCt^) method [50].

### 4.9. Statistical Analysis

Experimental results are presented as means with standard deviations (±SD), calculated from three independent experiments. Statistical analysis between treatment groups was conducted using one-way ANOVA followed by Tukey’s HSD post hoc test, with *p* < 0.05 considered statistically significant. All statistical analyses were performed using SPSS Statistics software (Version 22.0; IBM Corp., Armonk, NY, USA).

## 5. Conclusions

MeJA application enhanced flavonoid and phenolic compound accumulation while simultaneously triggering ROS oxidative stress, as demonstrated by increased H_2_O_2_ and O_2_^−•^ concentrations at 4 days post-germination. MeJA treatment upregulated enzymatic activities of key biosynthetic enzymes and elevated expression levels of associated genes while concurrently augmenting antioxidant enzyme activities. This investigation establishes that MeJA treatment effectively stimulates flavonoid biosynthesis in finger millet sprouts through modulation of the phenylpropanoid metabolic pathway and antioxidant defense mechanisms.

## Figures and Tables

**Table 1:** Instruments used in this study.

Instrument Name	Model	Manufacturer	Location (City, Country)
Intelligent light incubator	KM-68S	Ningbo Kemai Instrument Co., Ltd.	Ningbo, China
Thermostatic water bath	DK-S12	Shanghai Sumsung Laboratory Instrument Co., Ltd.	Shanghai, China
Electronic balance	JA2003	Cany Precision Instrument Co., Ltd.	Shanghai, China
UV-vis spectrophotometer	UV-1150	Shanghai Mapada Instruments Co., Ltd.	Shanghai, China
Desktop centrifuge	DICO	Anhui USTC Zonkia Scientific Instruments Co., Ltd.	Hefei, China
Real-time quantitative PCR system	StepOnePlus^TM^	Applied Biosystems (ABI)	Foster City, CA, USA
High-pressure steam autoclave	SX-500	TOMY Digital Biology Co., Ltd. (TOMMY)	Tokyo, Japan
High-speed refrigerated centrifuge	H1650	Changsha Xiangyi Centrifuge Instrument Co., Ltd.	Changsha, China

Note: All instruments were operated according to the manufacturers’ instructions.

**Table 2:** Primer sequences used for qRT-PCR analysis of relevant genes in finger millet. Reference gene: actin.

Name	Forward Primer (5′–3′)	Reverse Primer (5′–3′)
*Actin*	CTCACGCTCAAGTACCCAATC	GGCAACACGAAGCTCATTGTA
*EcPAL*	CGTGCCGCTCTCCTACATTGC	CCTCTGCTGCGTTCACCTTGG
*EcC4H*	GACTTCCGCTTCCTGCCGTTC	CACGAGCTTGCCGACGATGAG
*EcPOD*	CCAGGTGCTCTACTCCGACGACC	GAGGTTGGTCATGGCGGCGAC
*EcSOD*	CTCCTACGGCGACCTCTACCAGC	CTGAGGCTTGTCCTCCCTCCCTG
*EcAPX*	CACCTGTTCCTCGACTTTGC	TTACGTTGCAGCAGTTGAGG
*EcCAT*	ACCCGCCTTTACTACTTTTT	CATAGCCGAAAAGCATCCAT
*Ec4CL*	GACGACAAGGCGACCAAGGC	CTCCACGCTGCTGATGTTCTCG
*EcCHI*	GCCGCCGTGGAGAAGTTCAAG	ACCGACGAGTCCTTGGAGAACG
*EcCHS*	ATGCTGTTCTCCGTCCCGAATTTC	CTTATCTTCCTGGCGAGCACCTTC
*EcCHR*	AGTCTCAAGATCGCATTGCTGGTG	AACTTGTGGTGAGGTGTGCTGTG
*EcIFS*	AAGCAAGCGGATGTGGTGTTCTC	GCTCCACGTCACAGCCATATTCAG
*EcMYB*	AGGAGGAGGAAGATGCTGAAAGT	TTGAGGTGGTTGGATAGTGAGAG
*EcNAC*	CGTGTGCAAGGTGTTCAACA	CCAAGTAGTCGCTGAAGGAG

Note: CAT: catalase; SOD: superoxide dismutase; POD: peroxidase; APX: ascorbate peroxidase; PAL: phenylalanine ammonia-lyase; C4H: cinnamate 4-hydroxylase; 4CL: 4-coumarate: coenzyme A ligase; CHI: chalcone isomerase; CHR: chalcone reductase; CHS: chalcone synthase; IFS: isoflavone synthase; C4H: cinnamate 4-hydroxylase.

## Data Availability

The data presented in this study are available on request from the corresponding author.
